# Goat Whey Protein Hydrolysate Mitigates High-Fructose Corn Syrup-Induced Hepatic Steatosis in a Murine Model

**DOI:** 10.3390/nu17122011

**Published:** 2025-06-16

**Authors:** Chun-Hui Shao, Vipul Wayal, Chang-Chi Hsieh

**Affiliations:** 1 Department of Animal Science and Biotechnology, Tunghai University, Taichung 407224, Taiwan; alex.ppp001122@gmail.com; 2 Department of Pharmacy, Central Clinic & Hospital, Taipei 106441, Taiwan

**Keywords:** goat whey protein hydrolysate, hepatic steatosis, high-fructose corn syrup, bioactive peptides, ketohexokinase, fatty acid synthase

## Abstract

**Background/Objectives**: Hepatic steatosis, characterized by abnormal fat accumulation in the liver, is a major health concern with limited effective treatments. Goat milk whey proteins have demonstrated various therapeutic benefits. This study aimed to evaluate the hepatoprotective effects of goat whey protein hydrolysate (GWPH) on high-fructose corn syrup (HFCS)-induced hepatic steatosis in a murine model. **Methods**: The GWPH was prepared through enzymatic hydrolysis using Alcalase^®^ and divided into fractions: GWPH03 (<3 kDa), GWPH0310 (3–10 kDa), GWPH1030 (10–30 kDa), and GWPH30 (>30 kDa). These fractions were administered to respective GWPH treatment groups at 200 mg/kg b.w/day via intragastric gavage for 8 weeks, with HFCS provided to all groups except the Naïve group. After dietary intervention, an oral glucose tolerance test (OGTT) was performed, and the mice were then sacrificed for further analysis. **Results**: Our results demonstrate that GWPH mitigates HFCS-induced hepatic steatosis, reduces body weight gain, improves glucose homeostasis, alleviates liver injury, and regulates hepatic lipid metabolism. Notably, GWPH treatment significantly suppressed hepatic fatty acid synthase (FASN) expressions, indicating reduced de novo lipogenesis (DNL). Molecular docking of the identified peptides from GWPH—particularly PFNVYNVV, which showed strong binding affinity for KHK—suggests that it has potential as a competitive inhibitor of fructose metabolism. **Conclusions**: Collectively, our findings suggest that GWPH and its derived peptides could be promising candidates for managing hepatic steatosis and related metabolic abnormalities.

## 1. Introduction

The liver is a vital metabolic organ involved in protein, fat, and carbohydrate metabolism. Impaired liver function can lead to chronic liver diseases, cirrhosis, liver cancer, and metabolic disorders. Among these, hepatic steatosis, or excessive fat accumulation in the liver, is a major health concern [1,2]. When liver fat accumulates and increases total liver weight by 5%, it is called hepatic steatosis or fatty liver, and most fatty livers are associated with metabolic syndrome, including obesity, diabetes, hyperlipidemia, and various cardiovascular abnormalities [3]. Hepatic steatosis affects approximately 25% of the global population, with prevalence rising due to increasing obesity and metabolic syndromes. It is particularly common in developed countries, where sedentary lifestyles and high-caloric diets contribute to its growing burden [4]. Fatty liver, previously known as non-alcoholic fatty liver disease (NAFLD), has been reclassified in 2023 as metabolic dysfunction-associated steatotic liver disease (MASLD) to emphasize its strong association with metabolic dysfunction [5]. Hepatic steatosis is graded by fat accumulation in hepatocytes: Grade 0 (<5%, normal), Grade 1 (5–33%, mild), Grade 2 (34–66%, moderate), and Grade 3 (>66%, severe) [4].

High fructose intake is strongly linked to hepatic steatosis due to its distinct hepatic metabolism. Unlike glucose, fructose bypasses key regulatory steps and is rapidly phosphorylated by ketohexokinase (KHK) to fructose-1-phosphate, leading to ATP depletion and unregulated entry into lipid synthesis pathways [6]. This accelerates hepatic de novo lipogenesis (DNL), where key lipogenic enzymes are notably upregulated, such as fatty acid synthase (FASN), thereby promoting excessive triglyceride (TG) accumulation and fat deposition in the liver. Excessive fructose consumption contributes to insulin resistance, oxidative stress, and inflammation, exacerbating hepatic lipid accumulation and increasing the risk of steatohepatitis and MASLD [7,8]. Given the central role of KHK in fructose metabolism, modulating its activity may provide a potential strategy for alleviating HFCS-induced hepatic steatosis and its associated metabolic complications [9]. Previous studies indicate that high-fructose corn syrup (HFCS) consumption further accelerates hepatic steatosis progression by promoting DNL and impairing metabolic homeostasis. Widely used in processed foods, soft drinks, and beverages, HFCS contains high levels of fructose and has been strongly associated with obesity and metabolic disorders. Notably, HFCS can induce metabolic syndrome-related alterations that are independent of weight gain, underscoring its role in the global rise of obesity, insulin resistance, and cardiovascular diseases [10,11,12].

Despite advancements in metabolic disease management, FDA-approved pharmacological treatments specifically targeting hepatic steatosis remain limited and are often associated with various adverse effects. Current therapeutic strategies largely emphasize lifestyle modifications, while existing pharmacological options, such as insulin sensitizers and lipid-lowering agents, often have limited efficacy and potential adverse effects [13]. Given these challenges, there is a growing interest in functional food ingredients, including bioactive proteins, protein hydrolysates, bioactive peptides, and natural bioactive compounds with hepatoprotective properties. These functional food ingredients may exert hepatoprotective effects by multiple mechanisms, including the suppression of hepatic lipogenesis, enhancement of antioxidant defense systems, and reduction in chronic inflammation. Specifically, protein hydrolysate and bioactive peptides have been shown to activate cytoprotective pathways, such as the Nrf2 antioxidant pathway, and suppress pro-inflammatory signaling cascades, like NF-κB, thereby mitigating oxidative damage, inflammation, and lipid accumulation in the liver [14,15,16,17].

Goat milk, often considered a superior alternative to cow milk, has gained attention for its potential health benefits. Specifically, the whey proteins from goat milk have been found to offer numerous therapeutic advantages and health-promoting effects, including immunomodulatory effects, potential for allergy management, anti-inflammatory and antioxidant activities, and antimicrobial and anticancer properties [18]. A prior study has suggested that the enzymatic hydrolysis of whey proteins using proteases generates low-molecular-weight bioactive fractions, which may serve as potential candidates for mitigating conditions such as atherosclerosis and hepatic steatosis. Additionally, these whey protein hydrolysate fractions exhibit promising cardioprotective properties, highlighting their potential as novel functional ingredients for the management of cardiovascular diseases (CVDs) [19]. Furthermore, a recent study evaluated the in vitro biosafety and bioactivity of goat milk protein-derived hydrolysates, demonstrating their potential as functional ingredients. Casein and whey proteins were enzymatically hydrolyzed to generate gastrointestinal-stable peptides, which exhibited significant inhibitory activity against α-amylase, pancreatic lipase, and angiotensin-converting enzyme, along with antibacterial and immunomodulatory properties. Cytotoxicity analysis confirmed their safety, highlighting goat milk protein hydrolysates as promising candidates for nutraceutical and functional food applications [20].

However, a comprehensive evaluation of the impact of goat whey protein hydrolysate (GWPH) on hepatic steatosis remains lacking. Additionally, no studies have yet established a direct link between GWPH and hepatic steatosis. Therefore, this study aims to investigate the hepatoprotective effects of GWPH in a murine model of HFCS-induced hepatic steatosis and to elucidate its underlying mechanisms.

## 2. Materials and Methods

### 2.1. Chemicals and Reagents

Alcalase^®^ protease enzyme (≥2.4 U/g specific activity) and Rennet (≥4% protein) was purchased from Sigma-Aldrich Co. LLC. (St. Louis, MO, USA). A Pierce^®^ BCA Protein Assay kit was purchased from Thermo Fisher Scientific (Waltham, MA, USA). The HFCS was purchased from Fonen and Fonher Enterprise Co., Ltd. (Tainan City, Taiwan). A CareSens^TM^ II Blood Glucose Monitoring System purchased from i-SENS Inc. (Seoul, Korea). D-glucose was obtained from Sigma-Aldrich Co. LLC. (St. Louis, MO, USA). Alanine aminotransferase (ALT), aspartate aminotransferase (AST), triglycerides (TG), and total cholesterol (TC) Cobas assay kits were purchased from Roche Diagnostics Ltd. (Taipei, Taiwan). The anti-FASN rabbit polyclonal antibody (Catlog No. bs1498R) was purchased from Bioss Inc. (Woburn, MA, USA). An immunohistochemistry kit (Novolink Max Polymer Detection System) was purchased from Leica Biosystems Newcastle Ltd. (Newcastle Upon Tyne, UK).

### 2.2. Collection of Goat Milk Sample

Goat milk samples were obtained from Yi Jian Dairy Sheep Farm (Taichung City, Taiwan). Freshly collected milk was immediately chilled to 4 °C to prevent microbial growth and were transported to a laboratory under cold conditions.

### 2.3. Preparation of Goat Whey Protein (GWP)

Raw goat milk was pasteurized by indirect heating in a water bath at 62–65 °C with continuous stirring for 20 min. After pasteurization, the milk was cooled to 35 °C, and citric acid (3 g/L) was added to adjust the pH to approximately 4.0–5.0. The mixture was allowed to stand for 20 min, the rennet (0.4 g per 5 L of milk) was then added dropwise to further facilitate coagulation, and the mixture was next left undisturbed for 30 min at room temperature. After a 30 min incubation period, the coagulated curd was separated from the whey by filtration through muslin cloth. The resulting whey was collected and referred to as GWP. The protein concentration in GWP was determined using BCA protein assay following the manufacturer’s instructions.

### 2.4. Preparation of the Goat Whey Protein Hydrolysate (GWPH)

Enzymatic hydrolysis of GWP was performed using 1% Alcalase^®^ protease enzyme at 55 °C for varying durations (0, 1, 2, 4, and 6 h) to obtain GWPH. After hydrolysis, the reaction mixtures were filtered through glass wool to remove particulates and then centrifuged at 10,000× *g* for 30 min at 4 °C. The supernatants were collected and filtered sequentially through 0.45 µm and 0.22 µm membrane filters. Size-based fractionation was performed using centrifugal ultrafiltration tubes with molecular weight cut-offs of 30 kDa, 10 kDa, and 3 kDa, yielding the following hydrolysate fractions: GWPH03 (<3 kDa), GWPH0310 (3–10 kDa), GWPH1030 (10–30 kDa), and GWPH30 (>30 kDa). Each fraction was collected and stored at –20 °C until further analysis.

### 2.5. Identification of Bioactive Peptides from GWPH Using LC-MS/MS De Novo Sequencing

The peptide profiles of GWPH fractions (<3 kDa, 3–10 kDa, and 10–30 kDa) were analyzed using liquid chromatography–tandem mass spectrometry (LC-MS/MS). GWPH samples were desalted on a Zorbax 300SB C18 column (Agilent Technologies, Santa Clara, CA, USA) and then separated on a Waters BEH C18 column (1.7 μm, 100 μm × 10 cm) with a 70 min gradient of acetonitrile (0.1% formic acid) at 0.3 μL/min. MS analysis was conducted using an Orbitrap Elite ETD mass spectrometer (Thermo Fisher Scientific, Waltham, MA, USA). Peptide identification was performed using Proteome Discoverer (Mascot, UniProt) and de novo sequencing with PEAKS Studio v10.5 (Bioinformatics Solutions Inc., Waterloo, ON, Canada), with a 10 ppm precursor and 0.1 Da fragment ion tolerance. Peptides were filtered to FDR < 1% and ALC ≥ 50%. Fractions of <30 kDa were prioritized due to their higher abundance and bioactivity potential. As a result of this analysis, several bioactive peptides were identified from different molecular weight fractions of GWPH. Peptides from <3 kDa fraction demonstrated the highest relative abundance, with Lys-Tyr-Asp-Ser-Val-Leu-Ala-Val (KYDSVLAV) and Glu-Pro-Gln-Leu-His-Pro-Phe (EPQLHPF) representing 4.01% and 3.31%, respectively. In a 3–10 kDa fraction, Ala-Ser-His-Pro-Asp-Leu-Asn-Val-Val (ASHPDLNVV) and Thr-Pro-Val-Val-Val-Pro-Pro-Phe (TPVVVPPF) were identified with relative abundances of 3.03% and 2.84%, respectively. Peptides from the 10–30 kDa fraction, Pro-Phe-Asn-Val-Tyr-Asn-Val-Val (PFNVYNVV), exhibited a relative abundance of 2.52%. These peptides were selected for further investigation based on their prevalence, structural properties, and potential for bioactivity.

### 2.6. Animals and Experimental Design

A total of 60 six-week-old male C57BL/6JNarl mice were procured from National Laboratory Animal Center (NAR labs; Taipei, Taiwan). The mice were maintained on a 12 h light/dark cycle at a temperature of 22–25 °C and a humidity of 45–60% during a one-week acclimatization period. They were provided with a standard chow diet purchased from Altromin Spezialfutter GmbH & Co. KG (Lage, Germany) and distilled water ad libitum. After one week of acclimatization, the mice were weighed to record for initial body weight and were then randomly assigned into six groups (n = 10 per group). The Naïve group were fed with normal chow diet without any treatment and were provided with distilled water. The Control group received a standard chow diet supplemented with HFCS (30% *v*/*v*) in drinking water bottles along with sterile distilled water (10 mL/kg.bw/day) administered via intragastric gavage. The remaining four groups also received HFCS (30% *v*/*v*) in their drinking water bottles and were treated with different molecular weight fractions of GWPH at a dose of 200 mg/kg.bw/day. These groups included GWPH03 (GWPH fraction < 3 kDa), GWPH0310 (GWPH fraction 3–10 kDa), GWPH1030 (GWPH fraction 10–30 kDa), and GWPH30 (GWPH fraction > 30 kDa). The body weight of the mice was recorded weekly.

At the end of 8 weeks, the mice were kept for overnight fasting and the final body weight was recorded. Percentage body weight gain = final bw (g) − initial bw (g)/initial bw (g) × 100%. Then, the fasting blood glucose (FBG) test and oral glucose tolerance test (OGTT) were performed. Blood samples were collected, and the mice were euthanized by isoflurane inhalation. The livers and visceral white adipose tissues, including epididymal and perirenal white adipose tissues, were carefully dissected and weighed. The liver index was calculated as the liver weight (g)/final bw (g) × 100%. Animal experimental procedures were approved by the Institutional Animal Care and Use Committee (IACUC) of Tunghai University, Taiwan (ethical approval number: 102-020, 104-020).

### 2.7. FBG and OGTT

At the end of the 8th week of dietary intervention, the mice were fasted overnight. Blood was obtained from the caudal tail vein, and the FBG levels were measured using CareSens^TM^ II Blood Glucose Monitoring System. The FBG reading was considered as the ‘0-minute’ blood glucose level for the OGTT. After FBG measurement, the mice were administered D-glucose (2 g/kg. bw) via intragastric gavage, and the blood glucose levels were subsequently recorded at 30, 60, 90, 120, and 150 min. The area under the blood glucose curve (AUC) was then calculated to assess the glucose tolerance.

### 2.8. Determination of the Serum and Liver Biochemical Parameters

The serum levels of ALT and AST were measured using the UV spectrophotometric method with Cobas ALT and AST assay kits. The serum TG and TC contents were determined using the enzymatic colorimetric method with Cobas TG and TC kits. All assays were performed according to the manufacturer’s instructions. Similarly, the hepatic TG levels were measured in liver homogenates. The very low-density lipoprotein (VLDL) levels, defined as 1/5th of the TG level, in both serum and liver were calculated using the Friedewald formula [21,22].

### 2.9. Histopathological Examination

Fresh liver tissue specimens were fixed in a 10% neutral buffered formalin solution. The fixed tissues were then embedded in paraffin wax, sectioned into 5 µm slices, and stained with hematoxylin and eosin (H&E). The stained sections were examined under a Nikon ECLIPSE E200 microscope (Nikon Corporation, Tokyo, Japan), and photomicrographs were captured. The obtained photomicrographs were subsequently analyzed and compared to assess hepatic steatosis.

### 2.10. Immunohistochemical Staining

Paraffin-embedded liver tissue sections were deparaffinized, rehydrated, and subjected to antigen retrieval using citrate buffer (pH 6.0). Immunohistochemical staining for FASN expression was performed using the Novolink™ Max Polymer Detection System (Leica Biosystems). After antigen retrieval, the sections were incubated with an anti-FASN rabbit polyclonal primary antibody (1:100 dilution). The Novolink™ Polymer, an anti-rabbit Poly-HRP-IgG, was used as the secondary antibody. Visualization was achieved using 3,3′-diaminobenzidine (DAB) as the chromogen, which was followed by hematoxylin counterstaining. The FASN expression was evaluated under a Nikon ECLIPSE E200 microscope.

### 2.11. Molecular Docking Analysis of Bioactive Peptides from GWPH for KHK Inhibition

Molecular docking was performed, in accordance with a previous study by Wayal et al. (2025), to evaluate the binding affinity of the selected GWPH-derived peptides against KHK, a key enzyme involved in fructose metabolism [23]. The crystal structure of human KHK (PDB ID: 2HLZ) was obtained from the RCSB Protein Data Bank, with its structural details shown in Figure S6. Docking was performed using AutoDock 4.2 software [24], and binding interactions were visualized using BIOVIA Discovery Studio visualizer [25].

### 2.12. Statistical Analysis

Data are presented as the means ± SD. Multiple group comparisons were performed using one-way ANOVA, which were followed by Duncan’s post hoc test. A *p*-value of < 0.05 was considered statistically significant. All statistical analyses were conducted using SPSS 26.0 software (IBM, Chicago, IL, USA).

## 3. Results

### 3.1. Effect of GWPH on the Physiological Indicators in HFCS-Fed C57BL/6J Mice

After 8 weeks of HFCS feeding, the Control group exhibited significantly higher body weight, liver weight, and increased visceral adiposity in eWAT and prWAT compared to the Naïve group (*p* < 0.05, Table 1). Among the GWPH-treated groups for 8 weeks, GWPH1030 and GWPH30 significantly reduced body weight compared to the Control group (*p* < 0.05), while no significant changes were observed in the GWPH03 and GWPH0310 groups. Liver weights were significantly lower in all GWPH-treated groups compared to the Control group (*p* < 0.05). Visceral adiposity in eWAT and prWAT was significantly reduced in all GWPH treatment groups except GWPH03 (*p* < 0.05).

**Figure 1:**
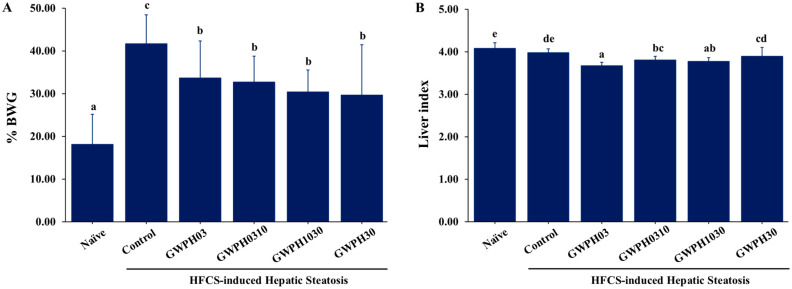
GWPH reduced the body weight gain and liver index in the HFCS-fed C57BL/6J mice: (**A**) percentage body weight gain; (**B**) liver index. Data are expressed as the mean ± SD (n = 10). Different superscript letters (a, b, c, d, and e) indicate significant differences between groups (*p* < 0.05).

As illustrated in Figure 1A, the Control group exhibited significantly higher body weight gain compared to the Naïve group (*p* < 0.05), whereas all GWPH-treated groups demonstrated a significant reduction in body weight gain compared to the Control group (*p* < 0.05). The liver index (wet liver weight-to-final body weight ratio) was elevated in the Control group, but with no significant difference from the Naïve group. However, the GWPH03, GWPH0310, and GWPH1030 treatments significantly lowered the liver index compared to the Control group, while GWPH30 did not show a significant difference (*p* < 0.05, Figure 1B).

### 3.2. Effect of GWPH on Serum Biochemical Parameters in the HFCS-Fed C57BL/6J Mice

**Figure 2:**
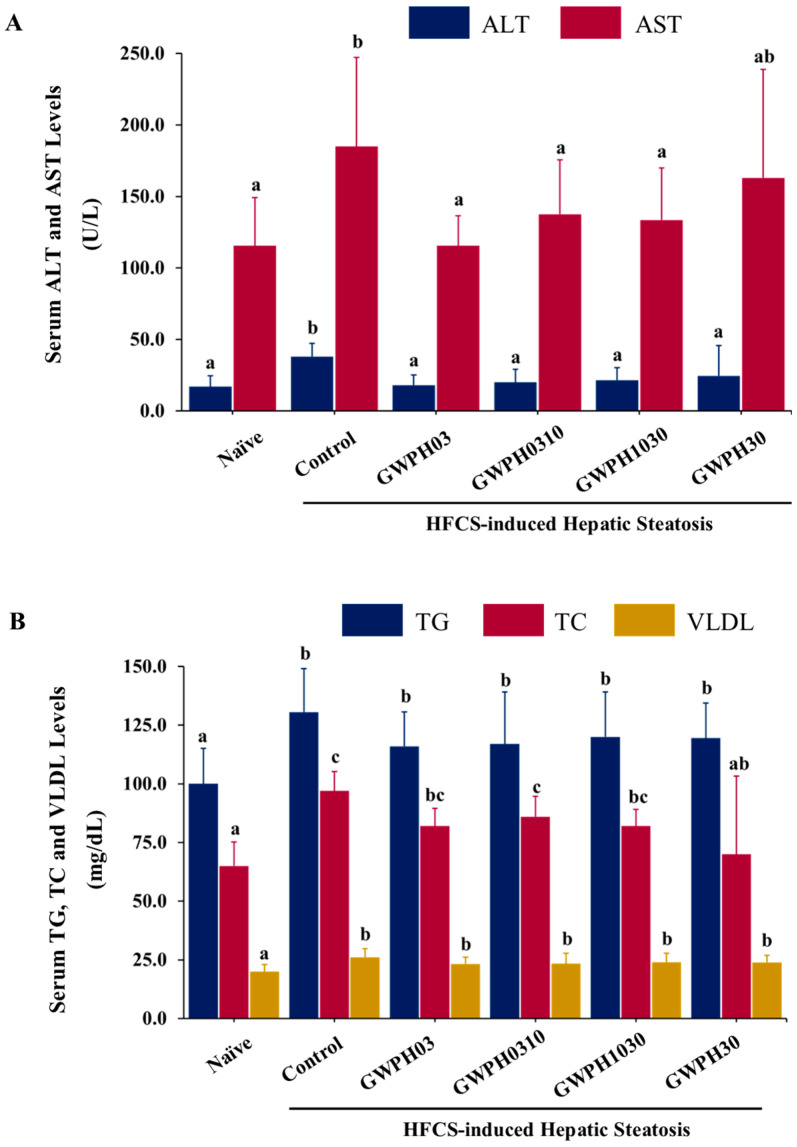
The effect of GWPH on serum biochemical parameters in the HFCS-fed C57BL/6J mice: (**A**) the serum levels of the liver injury markers: ALT and AST; (**B**) the serum lipid profiles: the TG, TC, and VLDL levels. Data are expressed as the mean ± SD (n = 10). Different superscript letters (a, b, and c) indicate significant differences between groups (*p* < 0.05).

Liver injury in the HFCS-fed C57BL/6J mice was assessed by measuring the serum levels of liver injury markers, including ALT and AST. In comparison to the Naïve group, the ALT and AST levels was significantly elevated in the Control group (*p* < 0.05). Notably, GWPH treatment significantly reduced these levels, showing a marked improvement in liver injury. The ALT and AST levels in GWPH-treated groups were significantly lower than in the Control group and showed no significant difference from the Naïve group (*p* < 0.05, Figure 2A), except for the AST levels in the GWPH30 group, which remained comparable to the Control group.

In the serum lipid profile, as shown in Figure 2B, the serum levels of TG, TC, and VLDL were significantly higher in the Control group compared to the Naïve group (*p* < 0.05). However, GWPH treatment did not significantly affect the serum TG, TC, or VLDL levels compared to the Control group, except for the GWPH30 group, which showed a significant reduction in TC levels (*p* < 0.05).

### 3.3. Effect of GWPH on Glucose Homeostasis in HFCS-Fed C57BL/6J Mice

**Figure 3:**
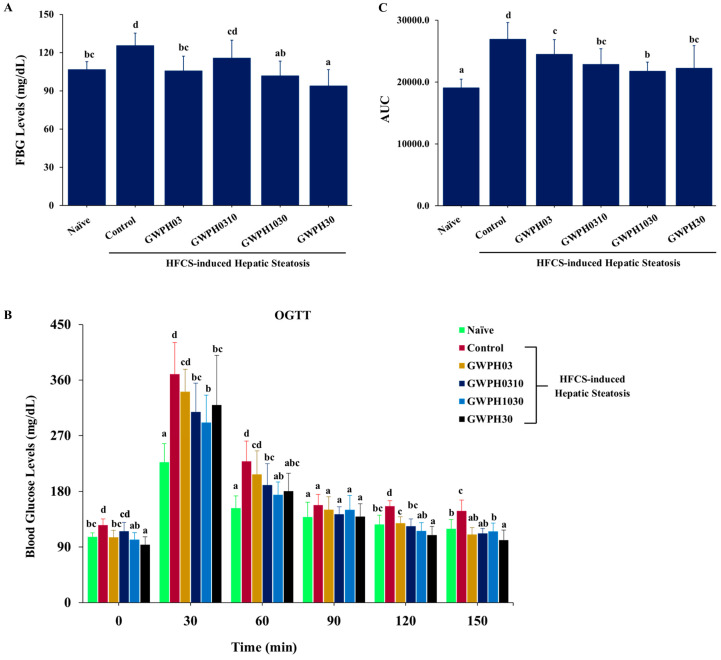
The effect of GWPH on glucose homeostasis in the HFCS-fed C57BL/6J mice: (**A**) FBG levels; (**B**) OGTT; and (**C**) the AUC of OGTT. Data are expressed as the mean ± SD (n = 10). Different superscript letters (a, b, c, and d) indicate significant differences between groups (*p* < 0.05).

Long-term HFCS consumption resulted in a significant elevation of FBG levels, impaired glucose tolerance, and an increased AUC for the OGTT in the Control group mice compared to the mice without HFCS feeding in the Naïve group (*p* < 0.05, Figure 3A–C). In contrast, GWPH dietary intervention for 8 weeks alongside HFCS intake significantly reduced the FBG levels in all treated groups, except for the GWPH0310 group, compared to the Control group (*p* < 0.05, Figure 3A). Additionally, all GWPH-treated groups showed improved glucose tolerance in the OGTT, as reflected by the lower glucose levels found when compared to the Control group (*p* < 0.05, Figure 3B). Moreover, GWPH treatment significantly decreased the AUC of the OGTT compared to the Control group, indicating a significant improvement in glucose homeostasis (*p* < 0.05, Figure 3C).

### 3.4. Effect of GWPH on Liver Lipid Levels and Hepatic Steatosis in the HFCS-Fed C57BL/6J Mice

**Figure 4:**
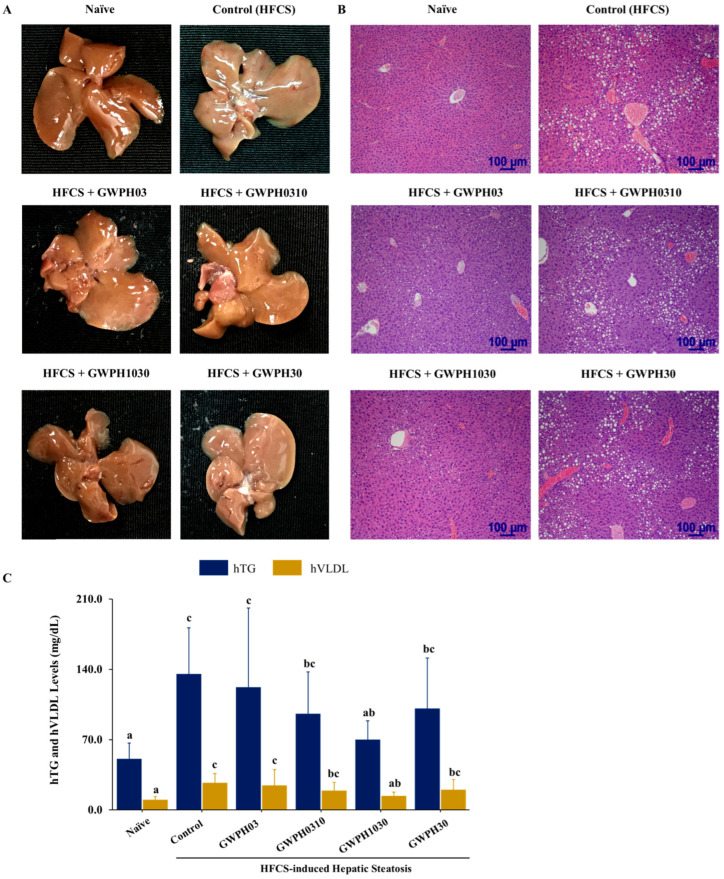
The effect of GWPH on hepatic lipid levels and hepatic steatosis in the HFCS-fed C57BL/6J mice: (**A**) liver gross morphology; (**B**) histopathological analysis of liver tissue using H&E staining (magnification: 100×, scale bar: 100 μm); and (**C**) hepatic lipid profile: hTG and hVLDL levels. Data are expressed as the mean ± SD (n = 10). Different superscript letters (a, b, and c) indicate significant differences between groups (*p* < 0.05).

Chronic intake of HFCS resulted in high fat accumulation and severe hepatic steatosis, as evidenced by the significant changes in liver gross morphology and histopathology in the Control group compared to the Naïve group (Figure 4A–C). However, GWPH treatment groups, including GWPH03 and GWPH1030, significantly alleviated hepatic steatosis and reversed the morphological alterations observed in the liver compared to the Control group (Figure 4A,B). As shown in Figure 4C, the Control group exhibited higher levels of hepatic TG and VLDL compared to the Naïve group (*p* < 0.05). In contrast, the GWPH-treated groups, particularly GWPH1030, demonstrated a significant reduction in hepatic TG and VLDL levels compared to the Control group (*p* < 0.05).

### 3.5. Effect of GWPH on Hepatic FASN Expressions in the HFCS-Fed C57BL/6J Mice

**Figure 5:**
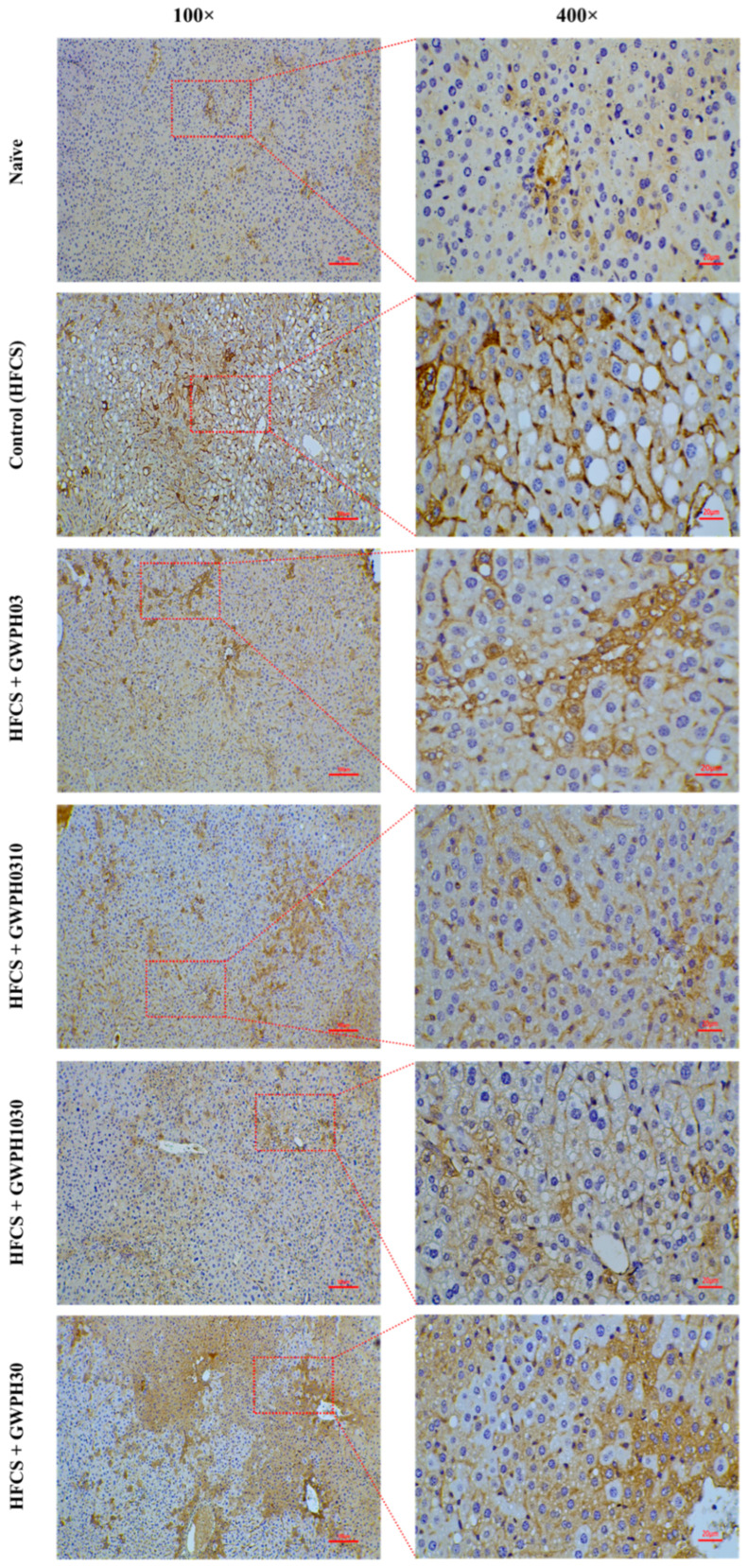
The effect of GWPH on hepatic FASN expressions in the HFCS-Fed C57BL/6J Mice: immunohistochemical analysis of the hepatic FASN expressions at 100× (scale bar: 100 μm) and 400× (scale bar: 20 μm) magnification.

Immunohistochemical analysis revealed that chronic HFCS intake markedly increases hepatic FASN expression, as indicated by the strong cytoplasmic staining in the Control group compared to the Naïve group (Figure 5). In contrast, GWPH treatment for eight weeks significantly attenuated FASN expression, with reduced staining intensity observed in the liver sections compared to the Control group (Figure 5). Notably, the GWPH-treated groups, particularly GWPH1030, exhibited near-normal FASN expression levels, suggesting a protective effect of GWPH against HFCS-induced hepatic lipogenesis (Figure 5).

### 3.6. Molecular Docking of the Selected GWPH Peptides for KHK Inhibition

Five peptides (KYDSVLAV, EPQLHPF, ASHPDLNVV, TPVVVPPF, and PFNVYNVV) were selected from different molecular weight fractions of GWPH and were then evaluated for their inhibitory potential against KHK using molecular docking. The 3D structures of these peptides (Figures S1–S5) were prepared as ligands, while the crystal structure of KHK (PDB ID: 2HLZ) was used as the target protein, both of which were prepared in docking PDB format using BIOVIA Discovery Studio. The results were compared with those reported by Zhu et al. (2023), wherein Compound **14** (KHK-IN-4) was identified as a potent KHK inhibitor [26]. The docking of Compound **14** with KHK served as a reference docking in the present study.

**Figure 6:**
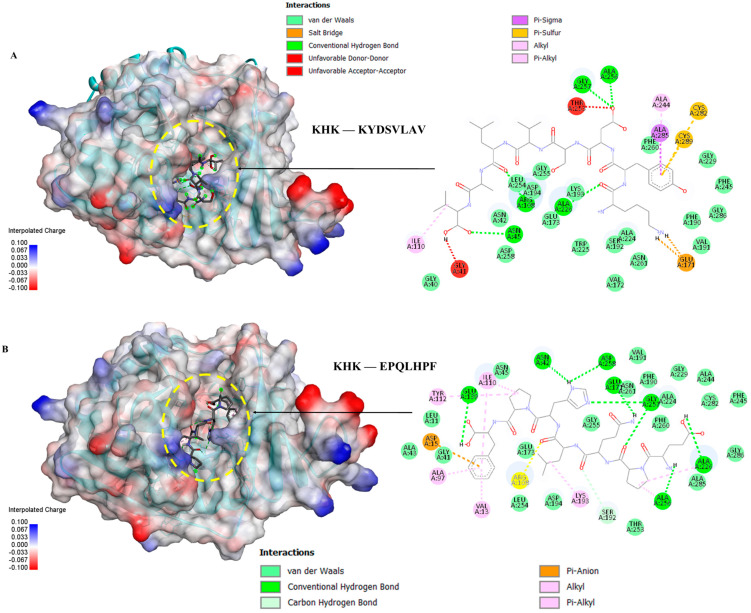
The molecular docking of peptides KYDSVLAV and EPQLHPF with KHK: (**A**) the binding pose and docking interactions of KYDSVLAV with KHK; (**B**) the binding pose and docking interactions of EPQLHPF with KHK.

**Figure 7:**
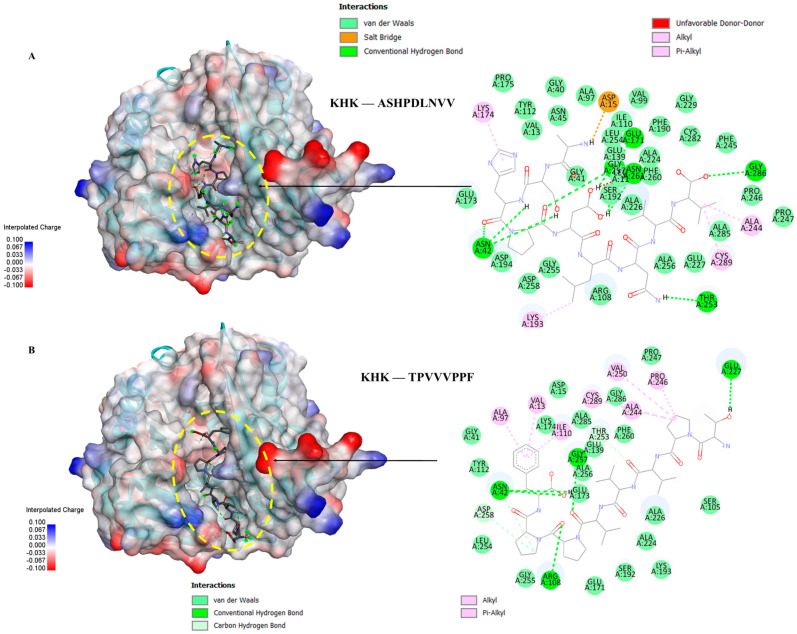
The molecular docking of peptides ASHPDLNVV and TPVVVPPF with KHK: (**A**) the binding pose and docking interactions of ASHPDLNVV with KHK; (**B**) the binding pose and docking interactions of TPVVVPPF with KHK.

**Figure 8:**
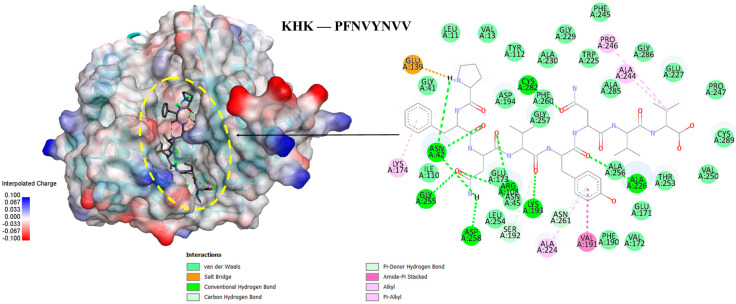
The molecular docking of PFNVYNVV peptide with KHK: the binding pose and docking interactions of ASHPDLNVV with KHK.

All five peptides exhibited distinct binding energies, docking poses, and key interactions with active site residues toward KHK (Table 2, Figure 6, Figure 7 and Figure 8). In principle, lower binding energies suggest stronger binding affinity. Notably, PFNVYNVV demonstrated the strongest binding affinity, with a binding energy of –9.6 kcal/mol, surpassing that of Compound **14**, which suggests a potentially more robust interaction. Moreover, PFNVYNVV displayed a docking pose and interaction profile that closely resembled that of Compound **14** (Table 2 and Figure 8), further underscoring its potential as a competitive KHK inhibitor.

## 4. Discussion

Hepatic steatosis, commonly known as fatty liver, is defined by the abnormal accumulation of lipids, particularly TG, within hepatocytes. It serves as a primary pathological feature of MASLD and is closely associated with the components of the metabolic syndrome [3,27]. Hepatic steatosis is a significant public health concern with limited therapeutic options and notable adverse effects, driving a growing interest in functional foods, such as bioactive proteins and peptides. Goat whey proteins are known for their therapeutic effects [18]. Therefore, this study aimed to investigate the hepatoprotective effects of GWPH in a HFCS-induced murine model of hepatic steatosis, with a focus on identifying potential bioactive peptides and elucidating their mechanisms of action.

Previous studies have shown that long-term HFCS consumption contributes to obesity, leading to increased body weight, fat accumulation, and elevated triglyceride levels [28,29]. In the present study, 8 weeks of HFCS feeding resulted in significant increases in body weight, liver weight, and visceral fat deposition in eWAT and prWAT (Table 1 and Figure 1). In contrast, the mice treated with GWPH alongside HFCS for 8 weeks demonstrated markedly reduced body weight gain, liver weight, and visceral adiposity. Therefore, our findings indicate that GWPH mitigates HFCS-induced obesity. These results are consistent with other studies that used protein hydrolysates derived from whey proteins [19], bovine α-lactalbumin [30], *Litopenaeus vannamei* waste [31], herring milt [32] and synthetic oligopeptides [22,23] in murine models of diet-induced obesity and fatty liver disease.

The hepatoprotective effect of GWPH was evaluated by assessing the serum levels of liver injury markers: ALT and AST. GWPH treatment significantly lowered these enzyme levels, indicating a notable improvement in liver function and attenuation of HFCS induced hepatic injury (Figure 2A). These results are consistent with a previous study by Wayal et al. (2025), where chicken meat hydrolysate demonstrated significant hepatoprotective effects against drug-induced liver injury [33]. Interestingly, the effects of GWPH on serum lipid profiles were less pronounced. GWPH treatment did not significantly alter the serum levels of TG, TC, and VLDL compared to the HFCS-fed control group. However, a significant reduction in TC levels was observed in the GWPH30 group, suggesting a selective lipid modulating effect (Figure 2A). Previous studies have indicated that impaired lipid exports, particularly reduced VLDL secretion, contribute to hepatic TG accumulation. This effect is exacerbated by enhanced de novo lipogenesis (DNL), ultimately promoting the development and progression of hepatic steatosis [34,35]. Consistent with these findings, our results indicate that GWPH treatment, especially the GWPH03 and GWPH1030 fractions, markedly attenuated hepatic steatosis and ameliorated liver histological alterations compared to the Control group (Figure 4A,B). Furthermore, as illustrated in Figure 4C, the Control group exhibited significantly elevated hepatic TG and VLDL levels. In contrast, the GWPH-treated groups, most notably GWPH1030, showed a significant reduction in hepatic TG and VLDL levels when compared to the Control group, suggesting an improvement in hepatic lipid metabolism and export.

T2DM is a crucial metabolic abnormality closely linked to hepatic steatosis, where they together exacerbate progression of MASLD [36]. Prior studies have reported that protein hydrolysates derived from black bean (*Phaseolus vulgaris* L.) [37] and pea protein [38] exhibit hypoglycemic effects by lowering fasting blood glucose levels and improving glucose tolerance in both human clinical trials and murine models of diet-induced diabetes. Similarly, camel milk protein hydrolysates have been shown to ameliorate hyperglycemia and hyperlipidemia in diabetic rats, suggesting their potent antihyperglycemic activity [39]. In line with these findings, our study showed that 8-week GWPH supplementation alongside HFCS intake significantly reduced FBG levels in all treatment groups, except GWPH0310 (Figure 3A). Additionally, the GWPH-treated groups demonstrated improved glucose tolerance during the OGTT, with lower blood glucose levels and a significantly reduced AUC compared to the Control group (Figure 3B,C), indicating enhanced glucose homeostasis.

**Figure 9:**
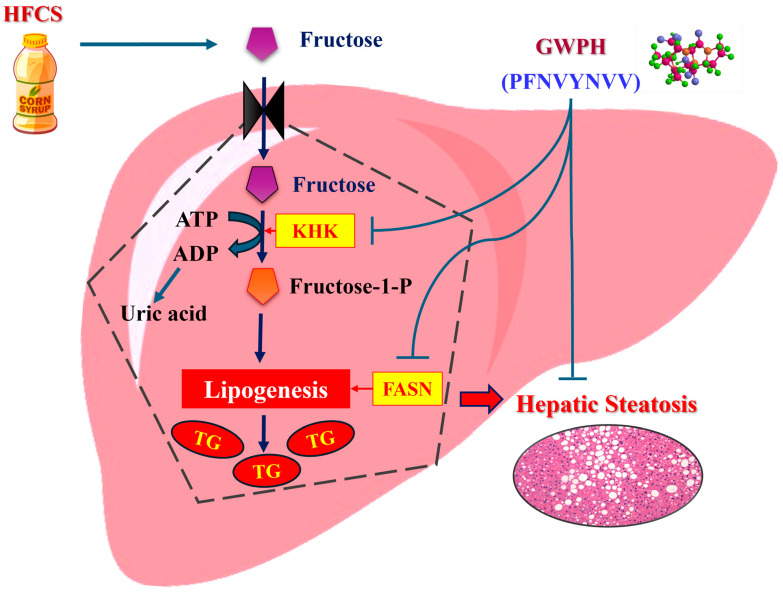
Schematic illustration of the proposed molecular mechanisms by which GWPH and its derived peptide (PFNVYNVV) mitigate HFCS-induced hepatic steatosis.

Consistent with the observed improvements in the hepatic lipid levels and steatosis, GWPH treatment markedly attenuated hepatic FASN expression in the HFCS-fed mice (Figure 5). In prior studies, it has been suggested that FASN is a key enzyme driving DNL, and its overexpression is understood to contribute to excessive hepatic TG accumulation under high-fructose conditions [40,41,42,43]. The reduction in FASN expression following GWPH administration suggests a direct inhibitory effect on hepatic lipogenesis, complementing its impact on upstream fructose metabolism (Figure 9). However, it is important to note that FASN expression was only assessed by IHC, which is a semi-quantitative method. The absence of Western blot or qPCR validation in this study represents a limitation that should be addressed in future research.

Excessive consumption of lipogenic sugars, such as fructose, contributes to the development of various metabolic disorders, including fatty liver disease [9]. Unlike glucose, fructose bypasses key regulatory steps and is rapidly phosphorylated by KHK to fructose-1-phosphate, resulting in ATP depletion and uncontrolled activation of lipid synthesis pathways. KHK is a critical enzyme in fructose metabolism, and its inhibition could be a potential therapeutic target for treating diet-induced metabolic disorders [6,26]. Prior in silico studies [44] and preclinical investigations using mouse and human liver models [45] have shown that the inhibition of KHK can reduce fructose-induced hepatic steatosis and alleviate its associated metabolic abnormalities. In line with these findings, our study identified five peptides (KYDSVLAV, EPQLHPF, ASHPDLNVV, TPVVVPPF, and PFNVYNVV) from various molecular weight fractions of GWPH and evaluated for KHK inhibitory potential via molecular docking. All five peptides exhibited distinct binding energies, docking poses, and key inter-actions with active site residues toward KHK (Table 2, Figure 6, Figure 7 and Figure 8). Notably, PFNVYNVV exhibited the strongest binding affinity (–9.6 kcal/mol), surpassing Compound **14** [26], and it mimicked its docking pose and interaction profile, suggesting its promise as a competitive KHK inhibitor.

While the >30 kDa (GWPH30) fraction was included in the in vivo study and showed protective effects, it was not analyzed by LC-MS/MS or molecular docking. This focus on <30 kDa fractions was based on their higher bioavailability and commonly reported bioactivity. However, the in vivo effects of GWPH30 suggest potential bioactivity, possibly from intact peptides or digestion-derived fragments. We acknowledge this as a study limitation and propose a future molecular characterization of GWPH30 to better elucidate its mechanisms. Moreover, although inflammation and immune cell infiltration are known contributors to steatosis progression, this study did not evaluate inflammatory markers or immune responses. Future investigations are warranted to determine whether GWPH modulates hepatic inflammation and immune cell recruitment, which could provide a more comprehensive understanding of its hepatoprotective mechanisms.

## 5. Conclusions

In summary, this study is the first to demonstrate that GWPH effectively attenuates HFCS-induced hepatic steatosis, mitigates body weight gain and visceral adiposity, alleviates liver injury, enhances glucose homeostasis, and regulates hepatic lipid metabolism in mice. Notably, peptides derived from GWPH, especially PFNVYNVV, exhibit strong binding affinity toward KHK, suggesting potential as competitive inhibitors of fructose metabolism (Figure 9). Importantly, GWPH treatment also significantly reduces hepatic FASN expression, indicating suppression of DNL and further supporting its protective effect against hepatic lipid accumulation. These findings highlight the therapeutic potential of GWPH and its peptides in managing hepatic steatosis and related metabolic disorders. However, future studies should focus on the direct evaluation of these peptides in vivo, investigate their molecular mechanisms, and validate KHK inhibition through in vitro enzymatic and structural analyses. Assessing their pharmacokinetics, bioavailability, and safety will also be crucial for advancing toward clinical application.

## Figures and Tables

**Table 1:** The effect of GWPH on physiological indicators in the HFCS-fed C57BL/6J mice.

Physiological Indicators	Naïve (Untreated)	HFCS-Induced Hepatic Steatosis				
		Control	GWPH03	GWPH0310	GWPH1030	GWPH30
Initial BW (g)	22.29 ± 1.25	22.48 ± 1.01	22.19 ± 1.56	22.30 ± 1.30	21.86 ± 0.94	22.18 ± 1.12
Final BW (g)	26.29 ± 1.46 ^a^	30.91 ± 1.90 ^c^	29.57 ± 2.05 ^bc^	29.56 ± 1.62 ^bc^	28.49 ± 1.42 ^b^	28.78 ± 3.25 ^b^
Liver wt. (g)	1.074 ± 0.091 ^a^	1.223 ± 0.076 ^b^	1.087 ± 0.091 ^a^	1.124 ± 0.047 ^a^	1.075 ± 0.060 ^a^	1.121 ± 0.142 ^a^
eWAT wt. (g)	0.366 ± 0.076 ^a^	1.004 ± 0.362 ^c^	0.801 ± 0.256 ^b^	0.736 ± 0.101 ^b^	0.724 ± 0.170 ^b^	0.729 ± 0.237 ^b^
prWAT wt. (g)	0.118 ± 0.072 ^a^	0.320 ± 0.085 ^c^	0.252 ± 0.103 ^bc^	0.233 ± 0.050 ^b^	0.185 ±0.049 ^ab^	0.231 ± 0.129 ^b^

BW, body weight; eWAT, epididymal white adipose tissue; and prWAT, perirenal white adipose tissue. Data are expressed as the mean ± SD (n = 10). Different superscript letters (a, b, and c) within the same row indicate significant differences between groups (*p* < 0.05).

**Table 2:** The binding energies and docking interaction profiles of the GWPH-derived peptides and Compound **14** with KHK.

Compound 14	KYDSVLAV	EPQLHPF	ASHPDLNVV	TPVVVPPF	PFNVYNVV
Δ*G* = −9.5 kcal/mol	−8.0 kcal/mol	−9.7 kcal/mol	−9.6 kcal/mol	−9.4 kcal/mol	−9.6 kcal/mol
ASN42	ASN42	ASN42	ASN42	ASN42	ASN42
ARG108	ARG108	ARG108	ARG108	ARG108	ARG108
GLU173	GLU173	GLU173	GLU173	GLU173	GLU173
SER192	SER192	SER192	SER192	SER192	SER192
LYS193	LYS193	LYS193	LYS193	LYS193	LYS193
ASP194	ASP194	ASP194	ASP194	-	ASP194
ALA224	ALA224	ALA224	ALA224	ALA224	ALA224
ALA226	ALA226	ALA226	ALA226	ALA226	ALA226
ALA230	-	-	-	-	ALA230
PHE245	PHE245	PHE245	PHE245	-	PHE245
PRO247	-	-	PRO247	PRO247	PRO247
VAL250	-	-	-	VAL250	VAL250
THR253	THR253	THR253	THR253	THR253	THR253
GLY255	GLY255	GLY255	GLY255	GLY255	GLY255
ALA256	ALA256	ALA256	ALA256	ALA256	ALA256
GLY257	GLY257	GLY257	GLY257	GLY257	GLY257
ASP258	ASP258	ASP258	ASP258	ASP258	ASP258
PHE260	PHE260	PHE260	PHE260	PHE260	PHE260
CYS282	CYS282	CYS282	CYS282	-	CYS282
ALA285	ALA285	ALA285	ALA285	ALA285	ALA285

Δ*G*, binding energy. Lower binding energies (or more negative values) indicate stronger binding affinity.

## Data Availability

The original contributions presented in this study are included in the article/Supplementary Material. Further inquiries can be directed to the corresponding author(s).

## Supplementary Materials

The following supporting information can be downloaded at: https://www.mdpi.com/article/10.3390/nu17122011/s1, Figure S1: 3D structure of the Lys-Tyr-Asp-Ser-Val-Leu-Ala-Val (KYDSVLAV) peptide; Figure S2: 3D structure of the Glu-Pro-Gln-Leu-His-Pro-Phe (EPQLHPF) peptide; Figure S3: 3D structure of the Ala-Ser-His-Pro-Asp-Leu-Asn-Val-Val (ASHPDLNVV) peptide; Figure S4: 3D structure of the Thr-Pro-Val-Val-Val-Pro-Pro-Phe (TPVVVPPF) peptide; Figure S5: 3D structure of the Pro-Phe-Asn-Val-Tyr-Asn-Val-Val (PFNVYNVV) peptide; Figure S6: Crystal structure of KHK (PDB ID: 2HLZ): Chains A, B, C, and D. Ketohexokinase (KHK). Resolution: 1.85 Å.

## Abbreviations

The following abbreviations are used in this manuscript:

HFCS	High-fructose corn syrup
GWPH	Goat whey protein hydrolysate
TG	Triglycerides
hTG	Hepatic triglycerides
TC	Total cholesterol
VLDL	Very low-density lipoprotein
hVLDL	Hepatic very low-density lipoprotein
ALT	Alanine aminotransferase
AST	Aspartate aminotransferase
FBG	Fasting blood glucose
KHK	Ketohexokinase
FASN	Fatty acid synthase
BCA	Bicinchoninic acid
BW	Body weight
eWAT	Epididymal white adipose tissue
prWAT	Perirenal white adipose tissue
