# Supplementary material for: Goat Whey Protein Hydrolysate Mitigates High-Fructose Corn Syrup-Induced Hepatic Steatosis in a Murine Model
Source: Nutrients. 2025 Jun 16;17(12):2011. doi: 10.3390/nu17122011 (PMC12196491; doi:10.3390/nu17122011)
Supplement: Supplementary file 1 [file nutrients-17-02011-s001.zip › nutrients-3650862-supplementary.pdf]

nutrients

Supplementary Materials

Goat Whey Protein Hydrolysate Mitigates High-Fructose Corn Syrup-Induced Hepatic Steatosis in a Murine Model

Chun-Hui Shao [1,2], Vipul Wayal [1,*] and Chang-Chi Hsieh [1,*]

[1] Department of Animal Science and Biotechnology, Tunghai University, Taichung 407224, Taiwan.
[2] Department of Pharmacy, Central Clinic & Hospital, Taipei 106441, Taiwan.
* Correspondence: wayalvipul9704@gmail.com (V.W); cchsieh@thu.edu.tw (C.-C.H)

Figure S1: 3D structure of Lys-Tyr-Asp-Ser-Val-Leu-Ala-Val (KYDSVLAV) peptide

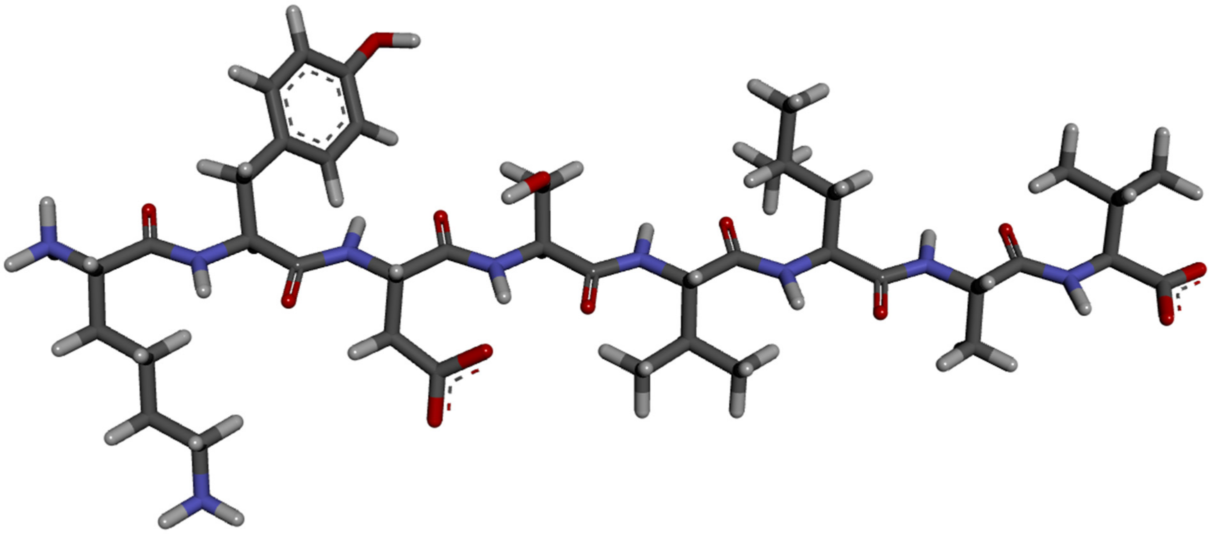

Figure S2: 3D structure of Glu-Pro-Gln-Leu-His-Pro-Phe (EPQLHPF) peptide

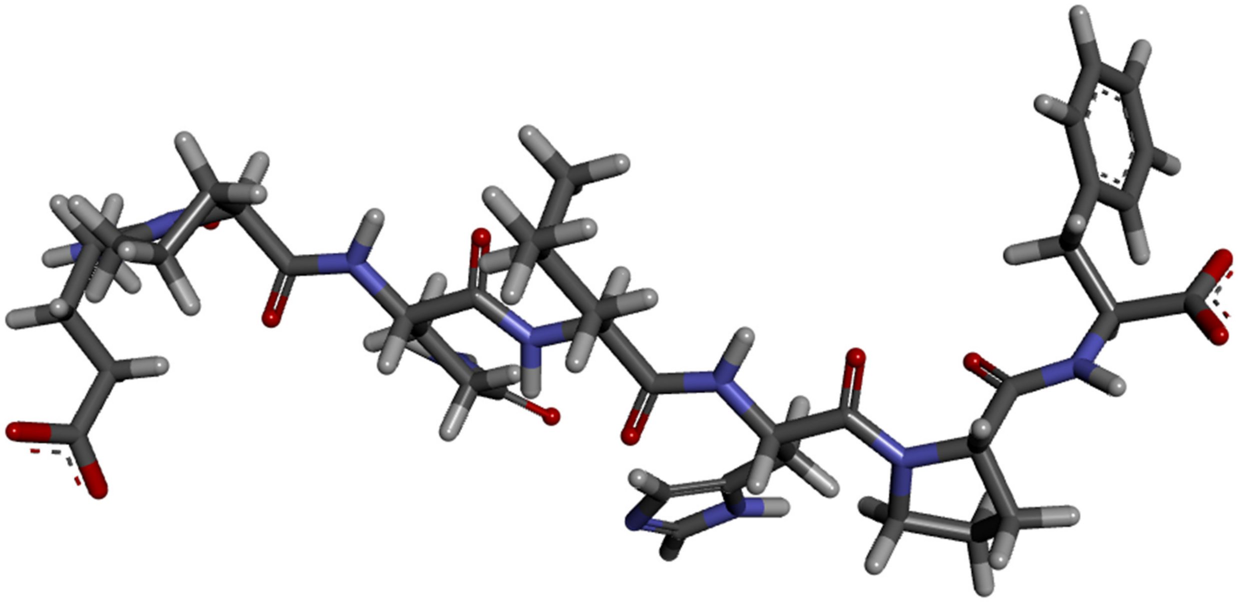

Figure S3: 3D structure of Ala-Ser-His-Pro-Asp-Leu-Asn-Val-Val (ASHPDLNVV) peptide

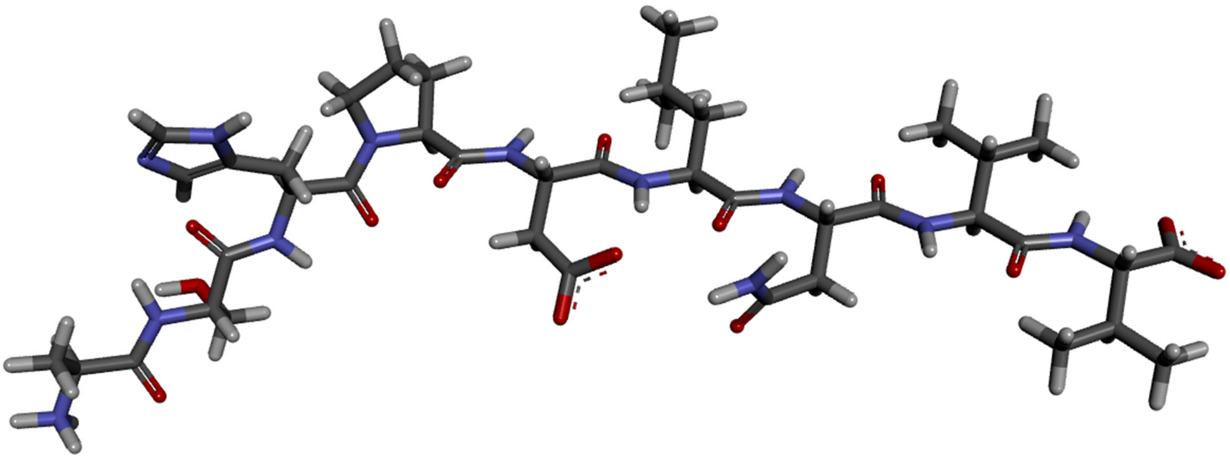

Figure S4: 3D structure of Thr-Pro-Val-Val-Val-Pro-Pro-Phe (TPVVVPPF) peptide

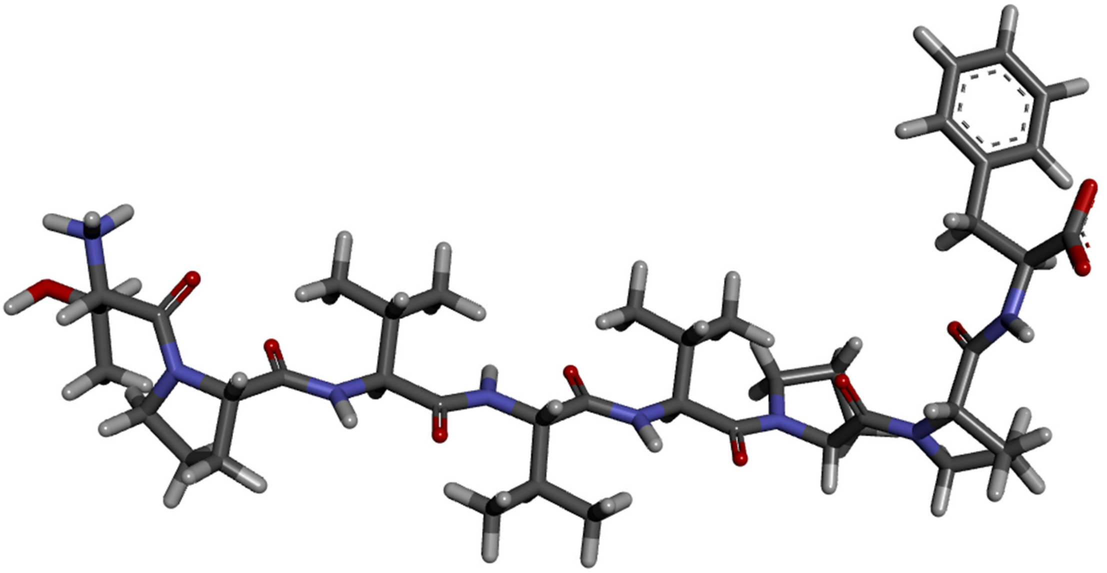

Figure S5: 3D structure of Pro-Phe-Asn-Val-Tyr-Asn-Val-Val (PFNVYNVV) peptide

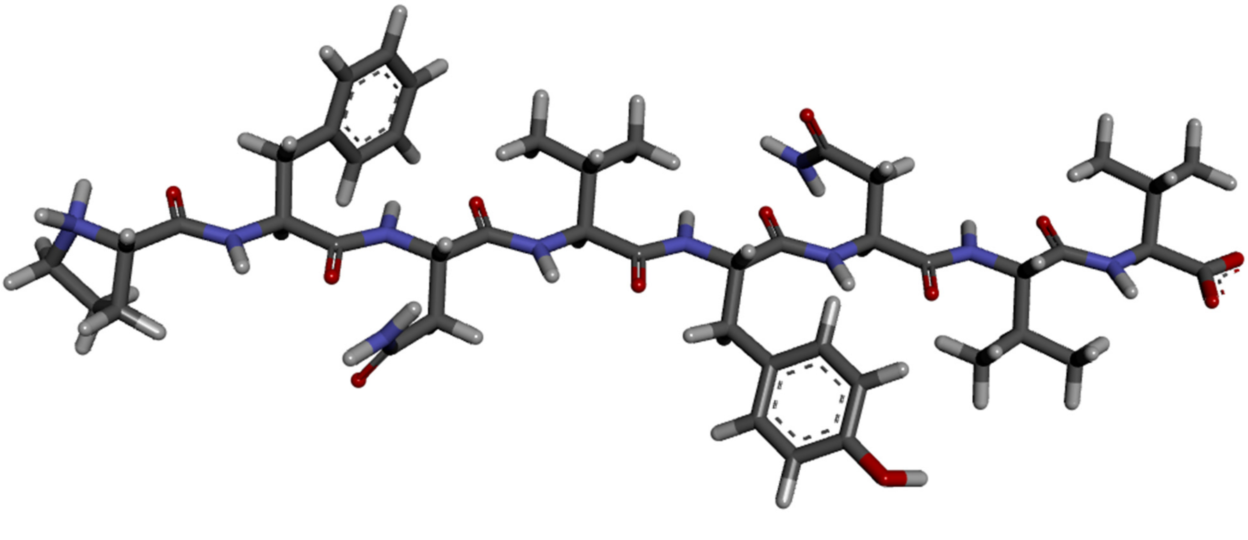

Figure. S6. Crystal structure of KHK (PDB ID: 2HLZ): Chains A, B, C, D: Ketohexokinase (KHK); Resolution: 1.85 Å

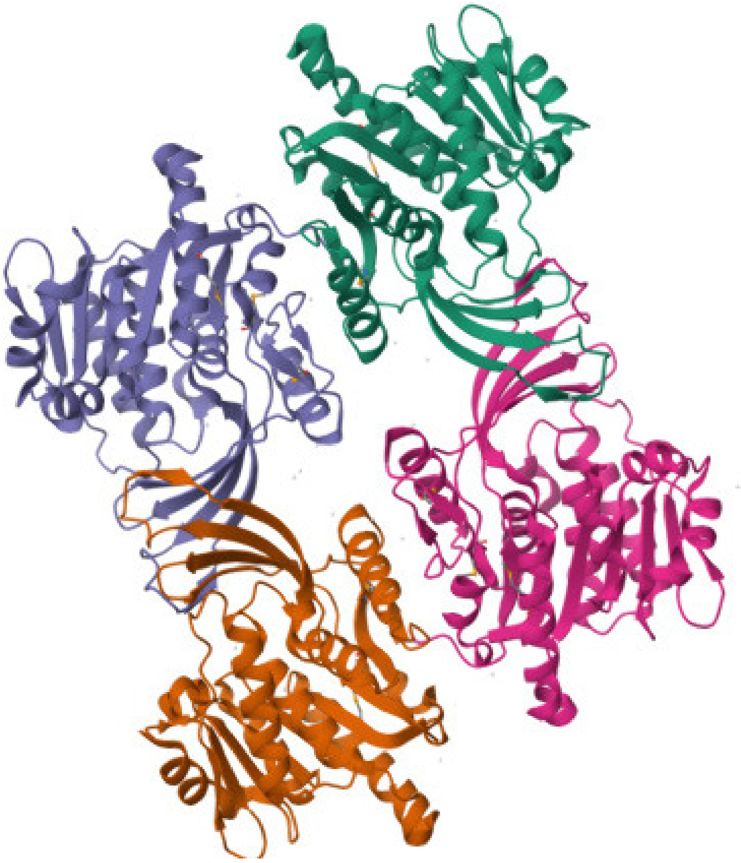

PDB DOI: https://doi.org/10.2210/pdb2HLZ/pdb
